# Comparison of the recovery of PCR-detectable porcine epidemic diarrhea virus and porcine reproductive and respiratory syndrome virus from filter papers under laboratory conditions

**DOI:** 10.3389/fcimb.2025.1677338

**Published:** 2025-10-22

**Authors:** Betsy Armenta-Leyva, Berenice Munguía-Ramírez, Danyang Zhang, Jianqiang Zhang, Rolf Rauh, Luis G. Giménez-Lirola, Jeffrey J. Zimmerman

**Affiliations:** ^1^ Department of Veterinary Diagnostic and Production Animal Medicine, College of Veterinary Medicine, Iowa State University, Ames, IA, United States; ^2^ Department of Statistics, College of Liberal Arts and Sciences, Iowa State University, Ames, IA, United States; ^3^ Tetracore Inc., Rockville, MD, United States

**Keywords:** environmental sampling, filter paper, PRRSV, PEDV, RT-qPCR

## Abstract

**Introduction:**

The need for cost effective surveillance of emerging human and veterinary pathogens has triggered a resurgence in research on environmental sampling methods, a process in which filter paper could play a role. The objective of this research was to compare the recovery of nucleic acids from paper products under laboratory conditions.

**Methods:**

In Experiment 1, commercially available paper products (n = 9) were saturated with water (1000 to 3000 µl) and the volume of decanted liquid measured and analyzed (linear regression). In Experiment 2, 4 paper products from Experiment 1 were evaluated for the release of RT-qPCR-detectable porcine reproductive and respiratory syndrome virus (PRRSV) RNA and porcine epidemic diarrhea virus (PEDV) RNA. Specifically, products were inoculated with PRRSV and PEDV, dried, subjected to 9 elution conditions (3 elution buffers × 3 soaking times), and tested by RT-qPCR. Thereafter, results were normalized and re-expressed as efficiency-standardized Cqs (ECqs).

**Results and Discussion:**

In Experiment 1, significant differences in recovery were observed across products and volumes (p < 0.05), with paper products 3 and 4 releasing the highest volumes. In Experiment 2, linear regression analysis showed that paper type, elution buffer, virus dilution, and their interactions affected viral RNA recovery (p < 0.05). AUC analysis showed no significant difference in PRRSV RNA detection between buffer-specific positive controls and product 3 eluted with lysis buffer or water. Similarly, no difference was detected in PEDV RNA detection between the positive control eluted with lysis buffer and products 3 and 4 eluted with lysis buffer. These results demonstrated that the choice of filter paper and the procedures used for viral RNA detection significantly affect target recovery.

## 1 Introduction

Environmental surveillance in the context of infectious diseases involves collecting and testing environmental samples (air, water, soil, or surfaces) for the purpose of detecting the presence of a specific pathogen. Environmental specimens may be collected using a variety of methods and technologies. For example, air samples may be actively collected by impingement, impaction, filtration, and electrostatic precipitation or passively collected using settling plates (Lidwell et al., 1983; Rahmani et al., 2020; Verreault et al., 2008). Water samples can be actively collected as grab samples of varying volumes or passively collected using diffusion chambers, absorptive devices, and filters (Adams et al., 2003; Matrajt et al., 2018; Shanmugam et al., 2024). And solid surfaces are commonly sampled using swabs, wipes, gauze, contact plates, sponges (Azeem et al., 2021; Jones et al., 2020; Turnage and Gibson, 2017).

The challenges posed by emerging and re-emerging human and veterinary pathogens and the need to detect and respond to their presence has triggered a resurgence in research on environmental sampling methods (Chia et al., 2020; Tiwari et al., 2023). This research has shown that, when combined with molecular detection methods, leave-in-place environmental sampling can provide efficient and effective surveillance. For example, cotton-based absorbent plugs or cotton gauze deployed in wastewater have been used to track severe acute respiratory syndrome coronavirus 2 (SARS-CoV-2) (Bivins et al., 2022; Cha et al., 2023; Corchis-Scott et al., 2021; Kevill et al., 2022). Cumulatively, these studies suggest that leave-in-place filter paper samplers could be a viable option for environmental surveillance in livestock populations.

The long-term objective of this research is the development of a practical, low-tech, low-cost, leave-in-place environmental sampling system for deployment in livestock production facilities. Such a system would be implemented by placing sampling devices in a production facility, e.g., a barn, collecting them at designated frequencies (e.g., weekly or monthly), and testing them for the pathogen(s) of interest. Filter papers are of particular interest in this application because of their relatively low cost, ready availability, and history of use in diagnostic medicine as a matrix for the collection, storage, and transport of biological samples. However, because paper products differ widely in composition and manufacture, it is reasonable to expect variation in the recovery of eluates and in the desorption of molecular targets. Thus, the objective of this research was to compare the “laboratory compatibility” of commercially available filter paper products (n = 9) in terms of liquid recovery (Experiment 1) and then evaluate a subset of these (n = 4) for their ability to release RT-qPCR-detectable targets (Experiment 2).

## 2 Materials and methods

### 2.1 Experiment 1

**Figure 1:**
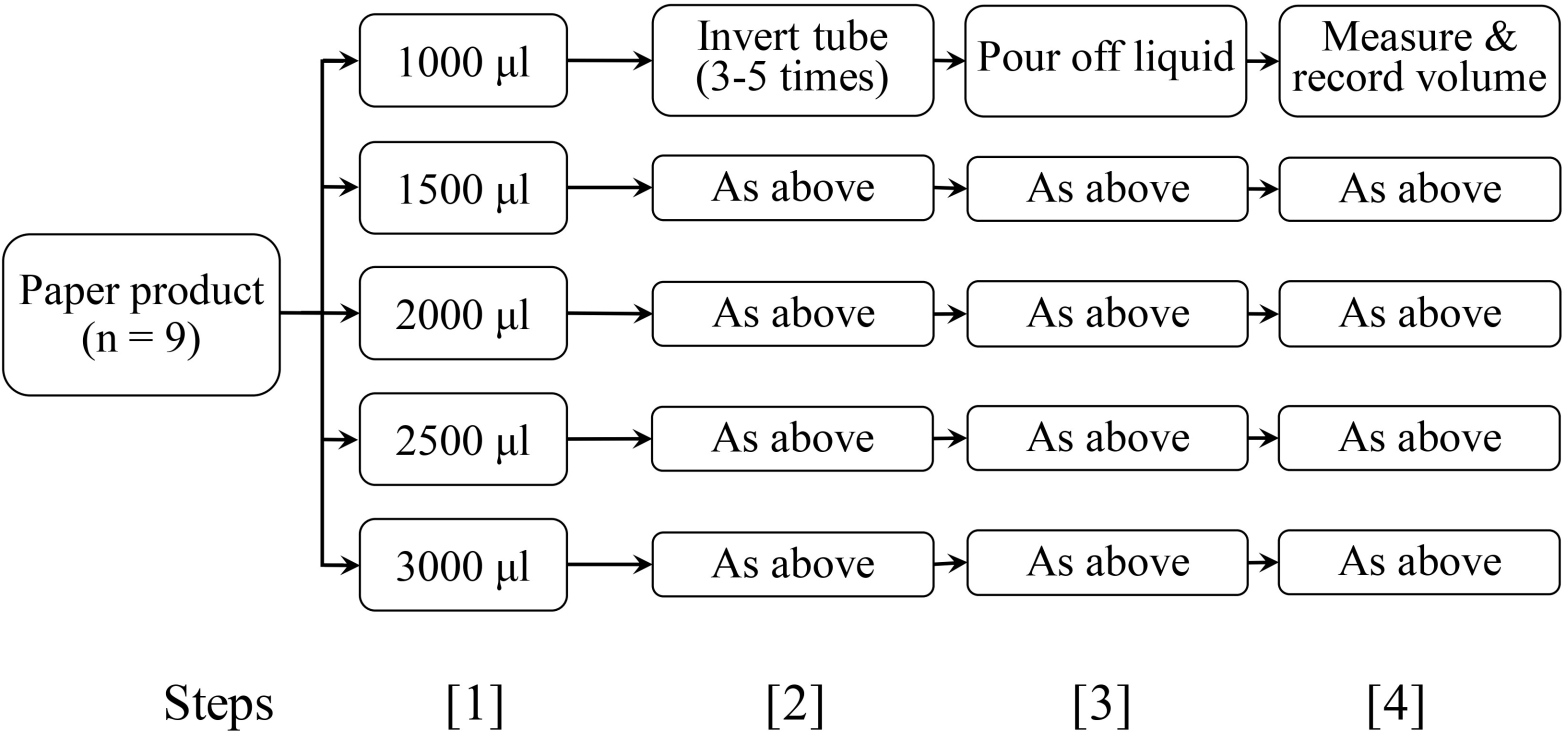
Procedure for Experiment 1 (liquid recovered from paper products after immersion): **1**. add a specific volume of PCR-grade water; **2**. invert the tube (3–5 times) to saturate the paper swatch; **3**. decant the free liquid into a clean tube; and **4**. measure and record volume recovered. Each combination of paper product and volume was tested in triplicate.

In Experiment 1, paper products (n = 9) were evaluated in terms of the volume of liquid recovered after immersion in a specific volume of water. As shown in **Figure 1**, after placing a paper swatch (5.1 cm × 3.8 cm; 2” × 1.5”) in a 50 ml conical tubes (Corning Life Sciences, Corning, NY), a specific volume of PCR-grade water was added (Step 1), tube manually inverted 3 to 5 times to saturate the paper (Step 2), then the free liquid was poured off into a clean 50 ml conical tube (Step 3) and the volume measured using a serological pipette (Step 4). A total of 135 samples were tested, i.e., the full factorial combination of 9 paper products and 5 volume levels with 3 replicates.

#### 2.1.1 Paper products evaluated in experiment 1

1. Whatman^®^ filter paper (Grade 903) – Constructed with a high percentage of alpha cellulose (98%). Commonly used for biological sample collection and analysis (catalog number: WHA10534612; Millipore Sigma, Burlington, MA, USA).
2. QIAcard™ FTA™ Classic cards – Cotton-based cellulose based paper treated with proprietary chemicals to lyse cells, denature proteins, and stabilize nucleic acids (catalog no. WHAWB120205; Millipore Sigma).
3. Reemay^®^ polyester filter media (Grade 2024) – Spunbond polyester matrix characterized by high strength and stability (catalog no. C79070FLT; Animal Care Systems; Centennial, CO, USA).
4. Swiffer^®^ dry cloth – Constructed from a nonwoven blend of polyester and polypropylene and intended for cleaning applications (catalog no. S-16029; Uline, Pleasant Prairie, WI, USA).
5. SmartSolve^®^ water-soluble paper (5 pt) – Primarily composed of wood pulp fibers and natural cellulose. Designed to dissolve and disperse upon contact with water (catalog no. IT120051; SmartSolve Industries, Bowling Green, OH, USA).
6. Dry surface filter polyester – Polyester-based air filter medium suitable for particle entrapment (catalog no. 2122K913; McMaster-Carr, Elmhurst, IL, USA).
7. Tacky surface filter polyester – Polyester matrix with a tacky surface to enhance its ability to capture particles (catalog no. 2122K274; McMaster-Carr).
8. Water-soluble starch foam – Primarily composed of biodegradable potato starch and designed to dissolve in water (catalog no. S-23846; Uline).
9. Sellars^®^ blue shop towels – Constructed of double re-creped (DRC) cellulose and intended for industrial cleanup applications (catalog no. 371781; Sellars, Milwaukee, WI, USA).

### 2.2 Experiment 2

**Figure 2:**
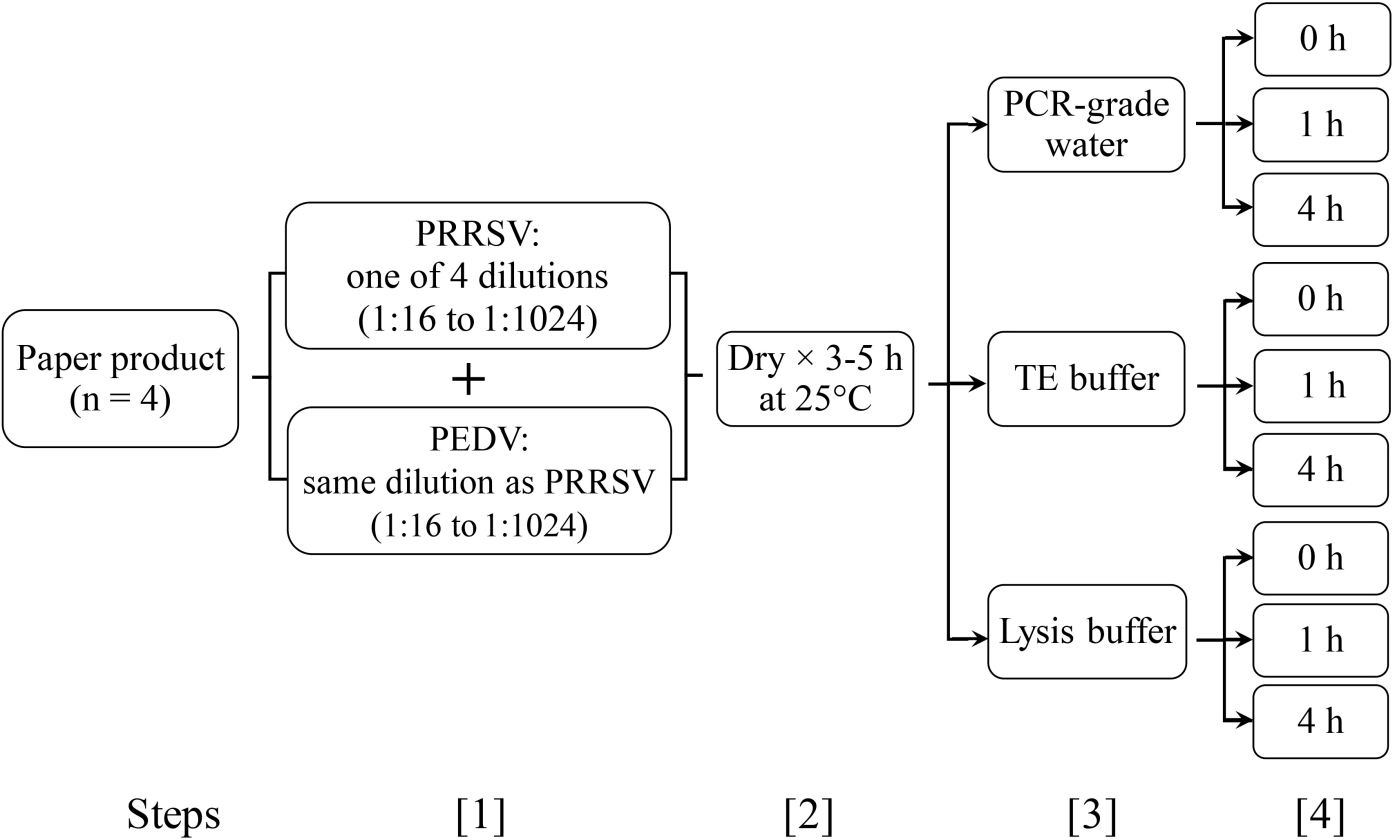
Procedure for Experiment 2 (recovery of PRRSV RNA and PEDV RNA): **1**. inoculate paper swatches with 300 μl of each virus at a specific dilution (1:16 to 1:1024); **2**. dry swatches (25°C incubator, 3 h - 5 h) and then store at -80°C in vacuum-sealed bags; **3**. Immerse swatch in PCR-grade water, Tris EDTA buffer, or general lysis buffer (Buffer G2, Qiagen); **4**. Recover eluate immediately (0 h) or hold at 4°C for 1 h or 4 h. Thereafter, store eluate at –80°C until tested. Each combination of paper product, elution buffer, and soaking time was tested in triplicate.

In Experiment 2, 4 paper products (1 to 4 in section 2.2.1) from Experiment 1 were inoculated with virus, dried, and then compared in terms of the recovery of porcine reproductive and respiratory virus (PRRSV) and porcine epidemic diarrhea virus (PEDV) RNA. Each paper product was evaluated under 9 elution conditions, representing all combinations of 3 elution buffers (PCR-grade water, Tris EDTA (TE) buffer, general lysis buffer), and 3 soaking times (0 h, 1 h, 4 h) (**Figure 2**).

#### 2.2.1 Virus

PRRSV isolate MN-184 (GenBank accession no. EF488739) was propagated on African green monkey kidney (MARC-145) cells to a concentration of 6 × 10^4^ median tissue culture infectious dose (TCID_50_) per ml using a protocol described elsewhere (Neat et al., 2021). PEDV isolate USA/NC35140/2013 (GenBank accession no. KM975735) was propagated in Vero cells (ATCC CCL-81) to a concentration of 1.7 × 10^5^ TCID_50_ per ml, as described elsewhere (Chen et al., 2014). This isolate was originally derived from piglet feces submitted to the Iowa State University Veterinary Diagnostic Laboratory (ISU VDL) for routine diagnostic testing (Chen et al., 2016).

#### 2.2.2 RT-qPCR reference standards

Reference standards were run on every RT-qPCR plate to provide for the normalization of RT-qPCR results. PRRSV reference standards were created by reconstituting a lyophilized 10-dose PRRSV modified live virus (MLV) vaccine (Ingelvac^®^ PRRSV MLV, Boehringer Ingelheim Vetmedica, Inc., Duluth, GA, USA) in 20 ml of PCR-grade water. This solution was then ten-fold serially diluted, and the dilution yielding a quantification cycle (Cq) value of ~30 (1:1000) was selected for use. PEDV reference standards were created from a commercial PEDV inactivated virus vaccine (Zoetis, Florham Park, NJ, USA). In brief, the vaccine was mixed well and 15 ml were centrifuged in a conical tube (Corning Life Sciences) at 3,300 × *g* for 30 min (IEC Centra CL3 centrifuge, Thermo Fisher Scientific, Inc, Waltham, Massachusetts). The supernatant was then ten-fold serially diluted and the dilution that achieved a Cq of ~30 was selected (1:10).

#### 2.2.3 Inoculation of paper products with virus

For inoculation of paper products, PRRSV and PEDV stock solutions were 4-fold serially diluted (1:16 to 1:1024) using PCR-grade water as diluent. Individual paper swatches (5.1 cm × 3.8 cm; 2” × 1.5”) were inoculated in 2 distinct spots with each virus at one dilution, e.g., 300 μl of PRRSV at a dilution of 1:16 in one spot and 300 μl of PEDV at 1:16 in a separate spot. For each of the 4 paper products, a total of 108 test swatches were prepared, i.e., 4 virus dilutions × 3 elution buffers × 3 soaking times × 3 replicates (**Figure 2**).

Negative control swatches (n = 3 per paper product) to monitor for cross-contamination were mock-inoculated with PCR-grade water and processed concurrently with test samples. Positive controls to establish the optimal viral RNA recovery were created by adding 300 μl of each viral dilution (1:16 to 1:1024) directly to 2 ml of the corresponding elution buffer. Because positive controls were created to mirror the test conditions of paper products, i.e., volume dilution of inoculum, and elution buffer), differences in RT-qPCR results between positive controls and test swatches represented the effect of paper. For each virus, a total of 36 positive controls were prepared, i.e., 4 virus dilutions × 3 elution buffers × 3 replicates.

Following inoculation, test swatches and negative control swatches were laid flat in a 25°C incubator (NAPCO 6301; Precision Scientific, Chicago, IL) until dry (3 h - 5 h), placed in vacuum-sealed bags (VacMaster^®^ Vacuum chamber pouches, and VacMaster^®^ VP215C, Ary, Inc., Kansas City, MO). Test swatches, negative control swatches, and positive controls were stored at -80°C.

#### 2.2.4 Recovery of viral RNA from paper products

For recovery of viral RNA, paper swatches were placed at 4°C (overnight), transferred to 50 ml conical tubes (Corning Life Sciences), and then one of 3 elution buffers was added to the tube (2 ml): PCR-grade water, TE buffer (Thermo Fisher Scientific, Inc.), or general lysis buffer (Buffer G2, Qiagen, Hilden, Germany). The tube was manually inverted (3–5 times) to ensure that the paper was saturated with the elution buffer. Three elution times were compared: 0 h, 1 h, and 4 h. Swatches eluted for 0 h were immediately decanted into a 5 ml tube (Corning Life Sciences) and stored at -80°C. Swatches eluted for 1 h or 4 h were held at 4°C in an environmental chamber (Percival Advanced Intellus Environmental Controller 130NLX, Percival Scientific, Inc, Perry, IA) and then decanted into 5 ml tubes and stored at -80°C.

#### 2.2.5 Extraction and RT-qPCR

Total nucleic acid extraction from sample eluates was done with the MagMAX™ Pathogen RNA/DNA Kit (Thermo Fisher Scientific, Inc.) on the KingFisher™ Flex automated extraction instrument (Thermo Fisher Scientific, Inc.) using a procedure described for low-cell content samples. In brief, the lysis/binding solution consisting of 125 μl of lysis/binding solution concentrate, 2 μl of carrier RNA, 125 μl of 100% isopropanol and 6 μl of the VetAlert™ inhibition control (IC) RNA Control (Tetracore Inc, Rockville, MD) was mixed with 100 μl of sample eluate and 20 μl of magnetic bead mix and then loaded into the extraction instrument. Purified nucleic acids (extracts) were eluted into 90 μl of MagMAX™ elution buffer. Every nucleic acid extraction plate included 86 research samples, 4 PRRSV reference standards, 4 PEDV reference standards, and 2 extraction controls (1 positive, 1 negative). Immediately thereafter, sample extracts were tested for nucleic acids. PRRSV RNA detection was done using the EZ-PRRSV™ MPX 4.0 Master Mix and Enzyme (Tetracore, Inc.) and PEDV RNA with the EZ-PED/TGE/PDCoV MPX 1.1™ (Tetracore, Inc.). Each RT-qPCR mixture was set up by combining 17.25 μl of EZ-PRRSV™ MPX 4.0 or PED/TGE/PDCoV MPX 1.1™ reagents, 0.75 μl of enzyme blend, and 7 μl of sample extract for a final reaction volume of 25 μl. Each plate included 1 positive and 1 negative amplification control, extracts from the 2 extraction controls, and extracts from the target-specific reference standards, i.e., 4 PRRSV reference standard extracts were included on PRRSV RT-qPCR plates and 4 PEDV reference standard extracts on PEDV RT-qPCR plates. Amplification of the VetAlert™ IC was observed on all samples tested, confirming the absence of inhibition. RT-qPCRs were run on the 7500 Fast thermal cycler (Applied Biosystems^®^) under the following conditions: 48°C for 15 m, 95°C for 2 m, and 45 cycles of 95°C for 5 s and 60°C for 40 s. Results were analyzed with the Sequence Detection Software (v1.5.1; Applied Biosystems^®^) and reported as raw Cqs.

#### 2.2.6 Normalization

Raw Cq values from PRRSV and PEDV RT-qPCRs were re-expressed as “Efficiency standardized Cqs (ECqs)” using Equation 1 (Armenta-Leyva et al., 2024; Pfaffl, 2001).


(1) $$\begin{array}{c} Efficiency\;standardized\;Cq\;\left(ECq\right)=E^{-\Delta Cq}=\\ E^{-(Sample\;Cq\;-\;Mean\;Cq\;of\;reference\;standards)} \end{array}$$

In Equation 1, E is amplification efficiency and ΔCq is the difference between a sample’s Cq and the mean Cq of the reference standards run on the same plate. Amplification efficiency can be expressed either as a ratio (number of target amplicons at the end of PCR cycle divided by the number at the beginning) or as a percentage. Thus, an efficiency of 2 or 100% would represent doubling at each cycle, i.e., perfect amplification. In this study, E values (ratios) for each reference standard were estimated from the raw fluorescence data using web-based software (LinRegPCR https://www.gear-genomics.com/rdml-tools), as described by Untergasser et al. (2021), and the arithmetic mean of the 4 reference standards E values was used in Equation 1. E estimates > 2 (i.e., 100%) were truncated at 2 to reflect the theoretical upper bound of exponential amplification, recognizing that E is inherently variable due to reaction kinetics and technical factors such as amplicon size, probe chemistry, plate material, and thermocycler performance as described elsewhere (Armenta-Leyva et al., 2024). ΔCq was calculated as the difference between the individual sample Cq and the arithmetic mean from the individual Cqs of the plate reference standards (n = 4). For samples with undetermined Cq values, a default Cq of 45 was assigned, corresponding to the total number of PCR cycles.

ECqs are interpreted as the fold change in the sample relative to the reference standard. For example, a sample with a Cq of 28 run on a plate with a reference standard mean E of 85% (E = 1.85) and mean Cq of 31 would have an ECq of 6.33 (ECq = E^-ΔCq^ = 1.85^–(28–31)^ = 6.33), that is, the concentration of target in the sample is 6.33 times the concentration of target in the reference standard.

#### 2.2.7 Analysis

Statistical analyses in Experiment 1 and 2 were conducted using RStudio v4.2.2 (RStudio Team, 2020). In Experiment 1, the volume recovered from paper swatches was expressed as the percent of the original immersion volume and the data were analyzed using a Type III ANOVA applied to a linear model that included paper product, immersion volume, and their interaction. To evaluate individual paper product performance, estimated marginal means (EMMs) were computed using the “emmeans” package in R (Lenth, 2022), and a contrast analysis with effect coding was applied to compare each paper’s recovery against the overall mean, with a Bonferroni adjustment to control for multiple comparisons.

In Experiment 2, cube root-transformed PRRSV and PEDV ECqs were used in all analyses. The transformed ECq responses for each pathogen were fitted to a linear regression model that included paper product, buffer, dilution, soaking time, and their two-way interactions as predictors. The analysis showed that soaking time and its interaction with other predictors were not significant (p = 0.91, Type III ANOVA) and, therefore, soaking time was removed from the model. Thus, the final linear regression model included paper product, buffer, dilution, and their two-way interactions as predictors and the positive control ECq response as the baseline.

To explain the overall effect of paper × elution buffer, the area under the curve (AUC) was calculated for each pathogen dilution series (paper × elution × replicate) using R package “MESS” (Ekstrøm, 2019) using the cube root-transformed ECq results. To assess the differences in detection using AUC as the response, a linear mixed-effects model was fitted (R “lme4” package; Bates et al., 2015) using paper, elution buffer, and their interaction as fixed effects and replicate as random effect. *Post-hoc* comparisons were performed using Tukey-adjusted estimated marginal means to identify paper products with AUC responses significantly different from the buffer-specific positive control.

## 3 Results

### 3.1 Experiment 1

A type III ANOVA showed that paper product (p < 0.05), immersion volume (p < 0.05), and their interaction (p < 0.05) affected the volume of liquid recovered after immersion (**Table 1**). A comparison of each paper’s overall recovery showed that paper products 3 and 4 had significantly higher recovery rates, while paper products 5 and 8 showed significantly lower recovery compared to the overall mean (*p* < 0.05).

**Table 1:** Recovery of liquid from paper products as a percent of the immersion volume (%, SEM).

Paper product	Immersion volume					
	1000 μl	1500 μl	2000 μl	2500 μl	3000 μl	Mean
1	28% (16.1)	31% (3.8)	48% (16.4)	70% (2.0)	68% (5.1)	49% (5.2)
2	15% (13.2)	29% (19.2)	50% (13.2)	56% (14.4)	61% (5.1)	42% (5.4)
3	63% (11.5)	67% (13.3)	77% (7.6)	85% (3.1)	89% (2.5)	76%* (3.3)
4	42% (16.1)	76% (10.2)	79% (7.2)	83% (4.6)	90% (3.3)	74%* (4.9)
5	- - - - no liquid recovered - - - -					0%* (0)
6	5% (5.0)	27% (23.4)	43% (7.6)	40% (8.0)	57% (6.7)	34% (5.4)
7	30% (20.0)	37% (11.5)	33% (11.5)	64% (6.9)	64% (5.1)	46% (4.9)
8	6% (3.8)	9% (10.2)	15% (13.2)	35% (20.5)	50% (16.7)	23%* (5.5)
9	17% (28.9)	13% (6.7)	30% (8.7)	72% (10.6)	53% (3.3)	37% (6.8)
				Overall mean		42% (7.9)

*Significantly different mean liquid recovery (%) relative to the overall mean (Type III ANOVA with Bonferroni adjustment).

### 3.2 Experiment 2

**Figure 3:**
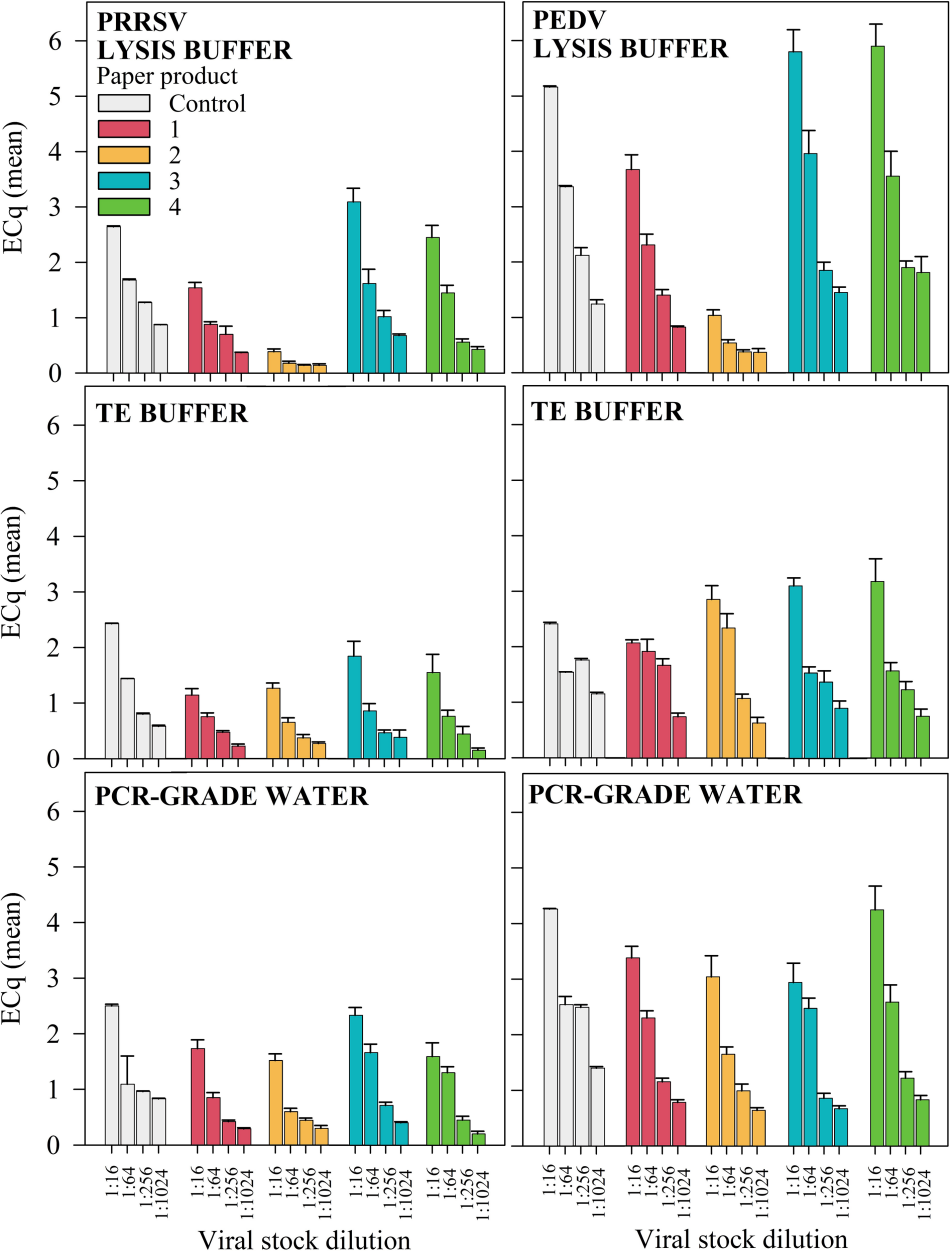
Experiment 2 - PRRSV and PEDV RT-qPCR results (ECqs; mean, SEM). Paper products were inoculated with 300 μl of viral stock dilution (1:16 to 1:1024), dried, and then eluted with 2 ml of general lysis buffer (buffer G2, Qiagen), Tris EDTA (TE) buffer, or PCR-grade water.

The performance of reference standards and amplification controls is summarized in **Table 2** to provide a point of comparison for Cq values once converted to ECqs, and to contextualize assay consistency across plates. A graphical representation of ECq responses by virus, virus stock dilution (1:16, 1:64, 1:256, 1:1024), controls, paper product, and buffer is given in **Figure 3**. Controls represent the optimal ECq responses because RNA extraction and amplification was directly from the virus stock solution and, therefore, provide a baseline of comparison. Linear regression modeling with a type III ANOVA revealed that paper product (p < 0.05), elution buffer (p < 0.05), dilution (p < 0.05), and their interactions (p < 0.05) significantly affected the ECq response for each virus (**Figure 3**).

**Table 2:** Performance summary of reference standards and amplification control across plates for PRRSV and PEDV RT-qPCR assays.

RT-qPCR	Plate	Reference standards			PAC		NAC	
		$\bar{\text{x}}$ Cq (range)	$\bar{\text{x}}$ ECq (range)	$\bar{\text{x}}$ E (range)	Cq	ECq	Cq	ECq
PRRSV	1	30.4 (29.3-31.0)	1.00 (0.86-1.19)	1.91 (1.88-2.00)	30.2	1.06	45.0	0.04
	2	30.9 (30.0-32.5)	1.00 (0.64-1.30)	1.87 (1.83-1.94)	29.9	1.21	45.0	0.05
	3	29.9 (29.8-30.1)	1.00 (0.95-1.03)	1.88 (1.86-1.90)	28.5	1.34	45.0	0.04
	4	29.3 (29.2-29.5)	1.00 (0.96-1.03)	1.88 (1.83-1.90)	28.2	1.27	45.0	0.04
	5	29.1 (29.1-29.2)	1.00 (0.98-1.02)	1.81 (1.78-1.85)	29.3	0.98	45.0	0.04
	6	29.1 (28.7-29.7)	1.00 (0.87-1.13)	1.81 (1.74-1.91)	28.7	1.10	45.0	0.04
PEDV	1	32.5 (30.9-34.3)	0.99 (0.62-1.62)	1.91 (1.89-1.93	31.1	1.37	45.0	0.07
	2	32.1 (31.2-32.9)	1.00 (0.78-1.27)	1.91 (1.83-2.00)	31.2	1.21	45.0	0.06
	3	31.6 (31.2-32.5)	1.00 (0.79-1.14)	1.87 (1.83-1.95)	30.6	1.25	45.0	0.04
	4	30.3 (30.2-30.5)	1.00 (0.95-1.01)	1.95 (1.88-1.99)	30.1	1.06	45.0	0.05
	5	31.1 (31.1-31.2)	1.00 (0.98-1.01)	1.87 (1.68-2.00)	30.5	1.13	45.0	0.05
	6	30.2 (30.1-30.3)	1.00 (0.96-1.01)	1.82 (1.76-1.84)	29.6	1.12	45.0	0.05

Reference standards (n = 4) were run on every RT-qPCR plate to provide for the normalization of RT-qPCR results. Cq = Quantification cycle; ECq = efficiency standardized Cq; E = mean amplification efficiency calculated from the reference standards included on each plate; PAC, positive amplification control; NAC, negative amplification control.

**Figure 4:**
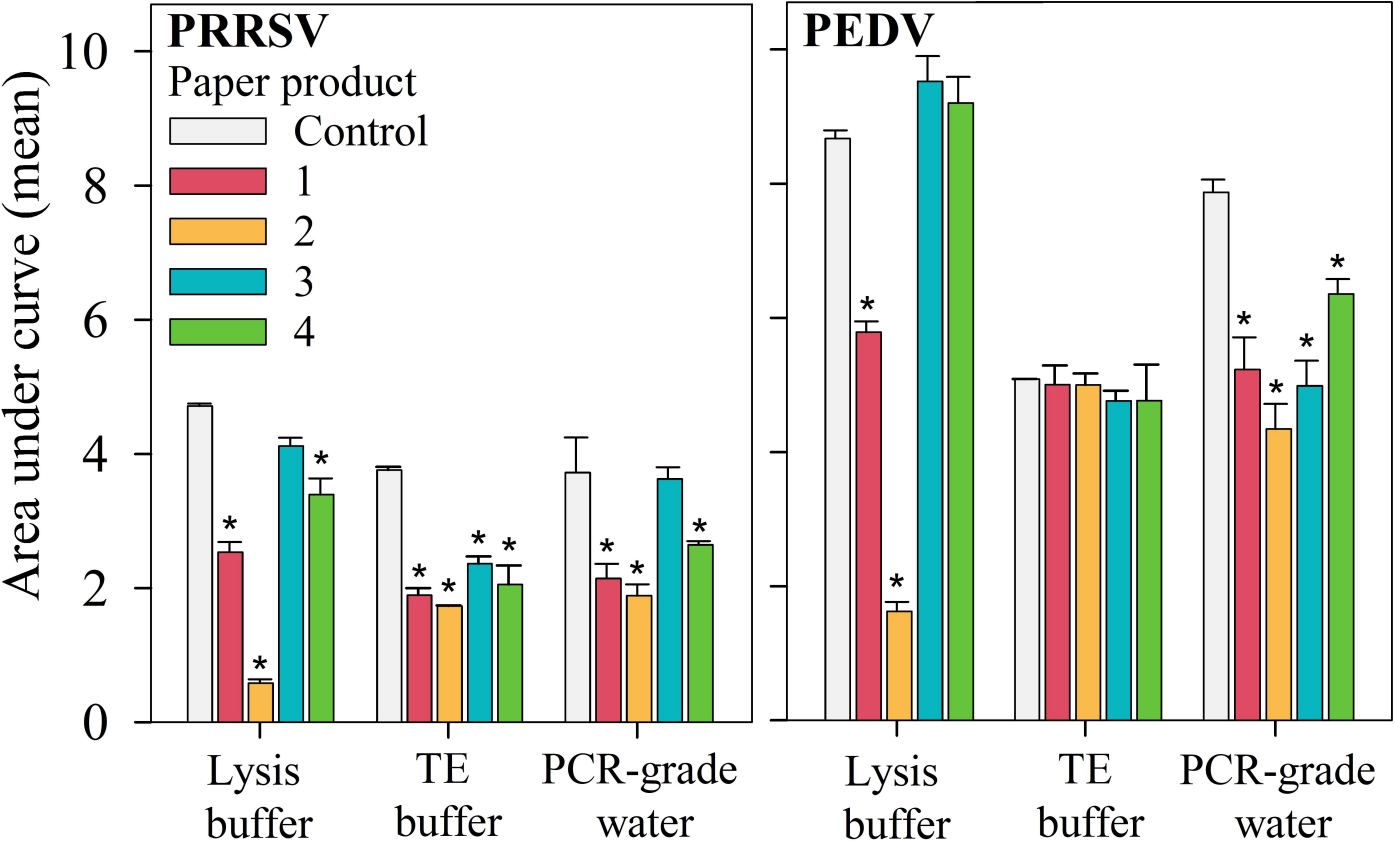
Experiment 2 - PRRSV and PEDV RT-qPCR results expressed as area under the curve (AUC; mean, SEM). Within elution buffers, differences in AUC (p < 0.05, linear mixed-effects model) between paper products and the respective positive control are indicated (*) (Section 2.2.7).

A graphical representation of AUC responses by buffer and paper product is provided in **Figure 4**. A linear mixed-effects model using AUC as the outcome showed that, among the PRRSV and PEDV positive controls, the lysis buffer yielded the largest overall mean AUCs. For PRRSV positive controls, the mean AUC for the lysis buffer were larger than the mean AUC for PCR-grade water (p < 0.05), but not TE buffer (p = 0.056). For PEDV positive controls, the lysis buffer control yielded a larger mean AUC than TE buffer (p < 0.05), but not PCR-grade water (p = 0.81).

A comparison of paper by buffer combinations with positive controls showed that, for PRRSV, the combinations of paper product 3 and lysis buffer or paper product 3 and PCR-grade water yielded AUC values that were not significantly different from their respective positive controls (p > 0.05). That is, under the conditions of this experiment, testing either of these combinations was comparable to testing the control itself. In the case of PEDV, paper products 3 and 4 paired with lysis buffer also produced AUCs comparable to the lysis buffer control (p > 0.05). All other combinations of paper and buffer provided lower performance, as measured by AUC.

## 4 Discussion

In both human and veterinary medicine, filter paper has long been used for sample collection, transport, and storage, particularly when cold-chain logistics are limited. During World War II, dried blood spots (DBS) collected on filter paper were used to test soldiers for *Treponema pallidum* antibody (Smit et al., 2014). Since the early 1960’s and up to the present, heel stick DBS and urine samples collected on filter paper have been used to test newborn babies for phenylketonuria (Guthrie and Susi, 1963). In addition, consistent with developments in diagnostic technologies, filter paper has also been used in conjunction with a variety of pathogen-specific nucleic acid assays, e.g., cytomegalovirus (Soetens et al., 2008), dengue virus (Prado et al., 2005), enteric adenovirus (Zlateva et al., 2005), Epstein-Barr virus (Imbronito et al., 2008), hepatitis B virus (Gupta et al., 1992), human immunodeficiency virus Type 1 (Masciotra et al., 2012), human papilloma virus (Gustavsson et al., 2011), human T-cell lymphotropic virus type 1 (Noda et al., 1993), Measles virus (Katz et al., 2002), Newcastle disease virus (Perozo et al., 2006), norovirus (Wollants et al., 2004), peste des petits ruminants virus (Hossain et al., 2017), PRRSV (Inoue et al., 2007), rabies virus (Picard-Meyer et al., 2007), rotavirus (Rahman et al., 2004), *Escherichia coli* (Mero et al., 2021), *Cryptosporidium* spp (Mero et al., 2021), *Haemophilus influenzae* (Peltola et al., 2010), *Leishmania* spp (Boggild et al., 2009), *Mycobacterium leprae* (Aye et al., 2011), *Plasmodium* spp (Zhong et al., 2018), *Salmonella* spp (Valiente Moro et al., 2007), *Trypanosoma* spp (Katakura et al., 1997).

While DBS is the most common filter paper specimen, other specimens used with filter papers include stool (Barua and Gomez, 1967; Wollants et al., 2004), oral fluids (Chibo et al., 2005; Mharakurwa et al., 2006), breast milk (Ayele et al., 2007), cervical smears (Gustavsson et al., 2011), cerebrospinal fluid (Peltola et al., 2010), skin smears (Aye et al., 2011), spleen aspirates, lymph node aspirate (Strauss-Ayali et al., 2004; El Tai et al., 2000), brain tissue smears (Rasolonjatovo et al., 2020), and lung tissue smear (Stringer et al., 2021). In addition to manually spotted diagnostic specimens, filter paper has also been utilized in leave-in-place (“passive”) environmental sampling, i.e., placing samplers in a given environment to passively capture target(s) over time (Górecki and Namieśnik, 2002; Kot-Wasik et al., 2007). For example, paper samplers placed in food processing areas have been used to detect the presence of *Listeria monocytogenes* (Bobal et al., 2019) and laboratory mouse cage monitoring studies have demonstrated the diagnostic utility of filter media deployed within individually ventilated cage systems. Reemay^®^ polyester filter media (paper product 3) has been implemented across various cage locations including the floor, bedding, exhaust ports and filter lids, enabling the detection of a variety of pathogens, including astrovirus, minute virus of mice, mouse hepatitis virus, mouse parvovirus, murine norovirus, Theiler murine encephalomyelitis virus, *Aspiculuris tetraptera*, *Entamoeba muris*, *Mycoptes musculinus*, *Myobia musculi*, *Radfordia affinis Spironucleus muris*, *Syphacia obvelata*, *Helicobacter* spp., *Klebsiella* spp., *Pasteurella pneumotropica*, *Proteus* spp., *Rodentibacter* spp (Dubelko et al., 2018; Gerwin et al., 2017; Hanson et al., 2021; O’Connell et al., 2021; Williams et al., 2024).

The 4 products considered in Experiment 2 (paper products 1, 2, 3, and 4) have previously been used in diagnostic or sampling applications. Paper product 1 (Whatman^®^ Grade 903) has been widely used for DBS samples to be tested for antibody and/or nucleic acid for a variety of pathogens, e.g. *Bordetella pertussis* (De Vitis et al., 2022), cytomegalovirus (Soetens et al., 2008), Ebola virus (Keita et al., 2018), hepatitis E virus (Singh et al., 2014), *Plasmodium falciparum* (Fuehrer et al., 2011), *Streptococcus pneumoniae* (De Vitis et al., 2022) and others. Similarly, FTA formats, including paper product 2 (QIAcard™ FTA™ Classic), have been widely used to transport and store nucleic acid for many pathogens, including *Mycobacterium leprae* (Aye et al., 2011), Newcastle disease virus (Perozo et al., 2006), *Pasteurella multocida* (Almeida et al., 2018), PRRSV (Inoue et al., 2007), rabies virus (Picard-Meyer et al., 2007), *Trypanosoma brucei* (Ahmed et al., 2011), West Nile virus, (Hall-Mendelin et al., 2010) and others. Paper product 3 (Reemay^®^ polyester filter media) is a polyester filter paper for use in mice cage monitoring systems (Dubelko et al., 2018; Gerwin et al., 2017; O’Connell et al., 2021; Williams et al., 2024). Lastly, paper product 4 (Swiffer^®^ dry cloth) is a commercial surface cleaning cloth that has been used in environmental sampling and detection studies involving pathogens such as African swine fever virus (Kwon et al., 2024), avian influenza virus (Azeem et al., 2021), PEDV (Phillips et al., 2022), PRRSV (Melini et al., 2024), *Salmonella* spp (Zewde et al., 2009), SARS-CoV-2 (Kotwa et al., 2021), and carbapenemase-producing bacteria (Too et al., 2024).

Appropriate to its intended application, the laboratory compatibility of any filter paper considered for use should be established. In this study, 9 commercially available filter paper products were screened for liquid recovery (Experiment 1). A subset (n = 4) with sufficient liquid recovery and prior use in diagnostic applications was evaluated for their ability to release RT-qPCR-detectable viral nucleic acids (Experiment 2). Reports in the literature highlight the importance of filter paper composition and elution chemistry in nucleic acid recovery. For instance, Zou et al. (2017) showed that DNA binding and release dynamics varied significantly across paper types, with untreated cellulose-based filter paper, e.g., Whatman^®^ Grade 1 and paper towels, outperforming other commercial blotting papers. Other studies have also demonstrated the importance of extraction conditions, including buffer composition, heating steps, fiber architecture, and the choice of nucleic acid extraction method (Elnagar et al., 2021; Hinlo et al., 2017; Koontz et al., 2015; Li et al., 2012; Sakai et al., 2014; Simon et al., 2020; Soetens et al., 2008). While our study focused on empirical performance under controlled conditions, prior work has demonstrated that material properties such as fiber type, pore size, and chemical surface modifications can influence target recovery (Kong et al., 2024; Tang et al., 2022). These mechanistic factors likely contribute to the observed differences in paper-buffer combinations, though they were beyond the scope of this study.

Under the controlled conditions described in this study, paper products 3 and 4 were the highest-performing. Indeed, recovery of PRRSV and PEDV was statistically equivalent to direct testing of the virus stock solution when paper product 3 was eluted with PCR-grade water or lysis buffer or, in the case of paper product 4, with lysis buffer. The use of these two products in environmental sampling has been previously described, e.g., environmental sampling in laboratory rodent colonies (paper product 3) or swabbing of environmental surfaces for PRRSV and PEDV (paper product number 4). Thus, our data are consistent with reports of pathogen recovery from these paper products and support their inclusion in further research on environmental surveillance applications. This study represents a laboratory-based screening effort to identify candidate materials for a low-cost, leave-in-place sampling system. While the experimental design was intentionally controlled to isolate paper performance under laboratory conditions, we acknowledge that field validation is necessary to assess real-world applicability.

## Data Availability

The raw data supporting the conclusions of this article will be made available by the authors, without undue reservation.
